# Editorial Comment: Erectile Dysfunction and Premature Ejaculation in Homosexual and Heterosexual Men: A Systematic Review and Meta-Analysis of Comparative Studies

**DOI:** 10.1590/S1677-5538.IBJU.2020.04.11

**Published:** 2020-05-20

**Authors:** Valter Javaroni

**Affiliations:** 1 Departamento de Andrologia Hospital Federal do Andaraí Rio de JaneiroRJ Brasil Departamento de Andrologia, Hospital Federal do Andaraí, Rio de Janeiro, RJ, Brasil

Barbonetti A ^1^, D’Andrea S ^2^, Cavallo F ^3^, Martorella A ^4^, Francavilla S ^4^, Francavilla F ^4^

^1^ Andrology Unit, Department of Clinical Medicine, Public Health, Life Sciences and the Environment, University of L’Aquila, L’Aquila, Italy; ^2^ Andrology Unit, Department of Clinical Medicine, Public Health, Life Sciences and the Environment, University of L’Aquila, L’Aquila, Italy; San Raffaele Sulmona Institute, Sulmona, Italy; ^3^ San Raffaele Sulmona Institute, Sulmona, Italy; ^4^ Andrology Unit, Department of Clinical Medicine, Public Health, Life Sciences and the Environment, University of L’Aquila, L’Aquila, Italy

J Sex Med. 2019 May;16(5):624-632

**DOI: 10.1016/j.jsxm.2019.02.014 | ACCESS: 10.1016/j.jsxm.2019.02.014**

## COMMENT

Dr. Arcangelo Barbonetti et al. published the first meta-analysis exploring the differences in the prevalence of ED and PE between homosexual and heterosexual men.

They found that homosexual orientation is associated with higher odds of erectile dysfunction (ED) and lower odds of premature ejaculation (PE) compared with heterosexual orientation. However, considering that only four studies could be included, the non-probabilistic nature of the samples and the use of different non-standardized indicators of sexual dysfunctions, their results should be interpreted with caution.

The fact is that homosexual individuals have been excluded from a significant number of important clinical trials. When dealing with non-heterosexual people, the investigation of sexuality is hindered by a methodological issue in that most of the questionnaires and diagnostic tools for the assessment of sexual disorders appear to be heterosexual oriented and have not yet been validated for homosexual populations ( 1 ).

Authors found that the discussed possible reasons why homosexual men have more chance of suffering with ED and multiple partners (less stability), a sense of competition and what they call: psychological stress - social stigmatization and discrimination against sexual minorities can jeopardize the psychological well-being of homosexuals ( 2 ).

The relationship between sexual orientation and ejaculatory function was controversial, since in three studies there is no significant association with sexual orientation. But inside meta-analysis, homosexual men exhibited a 28.0% lower odd of reporting PE compared with heterosexual controls. And is interesting that stable relationship (that was a protective factor in ED) here was a cause of sexual dysfunction. It has been suggested that the higher tendency of heterosexual men to engage in stable relationships might put them at a higher risk for PE compared with gay couples. Jern et al. ( 3 ) reported that the ejaculation latency time is negatively correlated to the duration of the relationship.

It is clear that we need more good quality comparative studies using validated tools to identify differences in sexual function/dysfunction among men with others sexual orientations.
